# A Rare Case of Maxillary Mucormycosis in a Child With Juvenile Diabetes Mellitus

**DOI:** 10.7759/cureus.61666

**Published:** 2024-06-04

**Authors:** Parmarth M Sonpal, Bhushan P Mundada, Nitin Bhola, Chetan Gupta

**Affiliations:** 1 Oral and Maxillofacial Surgery, Sharad Pawar Dental College and Hospital, Datta Meghe Institute of Higher Education and Research, Wardha, IND

**Keywords:** maxilla, juvenile, antifungals, diabetes, mucormycosis

## Abstract

*Mucor* and *Rhizopus* species are recognized as the primary culprits responsible for mucormycosis, a severe fungal infection known for its opportunistic nature. This infection primarily targets individuals with compromised immune systems, including those with diabetes mellitus and patients undergoing glucocorticoid therapy, where the immune response is weakened. This article aims to underscore the pivotal role of prompt diagnosis and intensive treatment in managing mucormycosis, particularly in pediatric patients, as it can avert death and mitigate serious morbidity. This case report emphasizes the urgency of identifying fungal infections in patients with diabetes early on and subsequently treating them aggressively to prevent adverse outcomes. It highlights the potential for excellent treatment outcomes when mucormycosis is promptly diagnosed and managed with intensive therapy. By doing so, significant morbidity and mortality associated with this condition can be effectively prevented, underscoring the importance of vigilance and proactive management in patients with predisposing factors for fungal infections.

## Introduction

Mucormycosis, also known as phycomycosis or zygomycosis, represents a formidable challenge in the realm of fungal infections, characterized by its severity and often devastating consequences. This invasive fungal disease is caused by organisms belonging to the class Zygomycetes, with *Mucor* and *Rhizopus* species emerging as the primary perpetrators. Classified as an opportunistic infection, mucormycosis primarily targets individuals with compromised immune systems, rendering them particularly susceptible to its insidious invasion. This includes patients who are immunocompromised due to various underlying conditions, such as uncontrolled diabetes mellitus, or those undergoing immunosuppressive therapies such as glucocorticoids [1]. The clinical spectrum of mucormycosis encompasses a diverse array of manifestations, with the literature delineating seven distinct types of the disease. Among these variants, rhinocerebral mucormycosis occupies a prominent position as the most prevalent form. This particular subtype exhibits a predilection for the facial skeleton, exerting its destructive influence on critical structures such as the sinuses, nasal mucosa, and surrounding tissues.

Mucormycosis has multifaceted modes of transmission, encompassing routes such as inhalation, ingestion, percutaneous entry, and implantation [2]. Once the insidious fungus infiltrates the body, it strategically targets vulnerable anatomical sites, including the nasal mucosa, sinuses, skin, and endothelium, laying the groundwork for its relentless progression.

In the context of rhinocerebral mucormycosis, the infection frequently initiates within the sinuses of the facial skeleton, encompassing key regions such as the ethmoidal, maxillary, and sphenoidal sinuses. From these anatomical footholds, the infection can rapidly propagate anteriorly, extending its sinister reach to encompass vital structures such as the maxilla and orbit. The clinical presentation of rhinocerebral mucormycosis is characterized by a constellation of symptoms, ranging from subtle to overt manifestations. These may include insidious signs such as tooth loosening, ulceration of the palate, nasal discharge, sinusitis, and facial pain, signaling the underlying pathological process. As the infection inexorably advances, it may precipitate more ominous complications, such as cellulitis, underscoring the urgency of early detection and intervention [2]. This case study offers a poignant illustration of the profound impact of mucormycosis, particularly in vulnerable populations. In this instance, a 14-year-old child afflicted with juvenile type 1 diabetes succumbed to mucormycosis affecting the maxilla [3]. This harrowing scenario serves as a stark reminder of the heightened vulnerability of individuals with diabetes to opportunistic infections such as mucormycosis, underscoring the imperative of meticulous management and timely intervention in such cases.

## Case presentation

**Figure 1:**
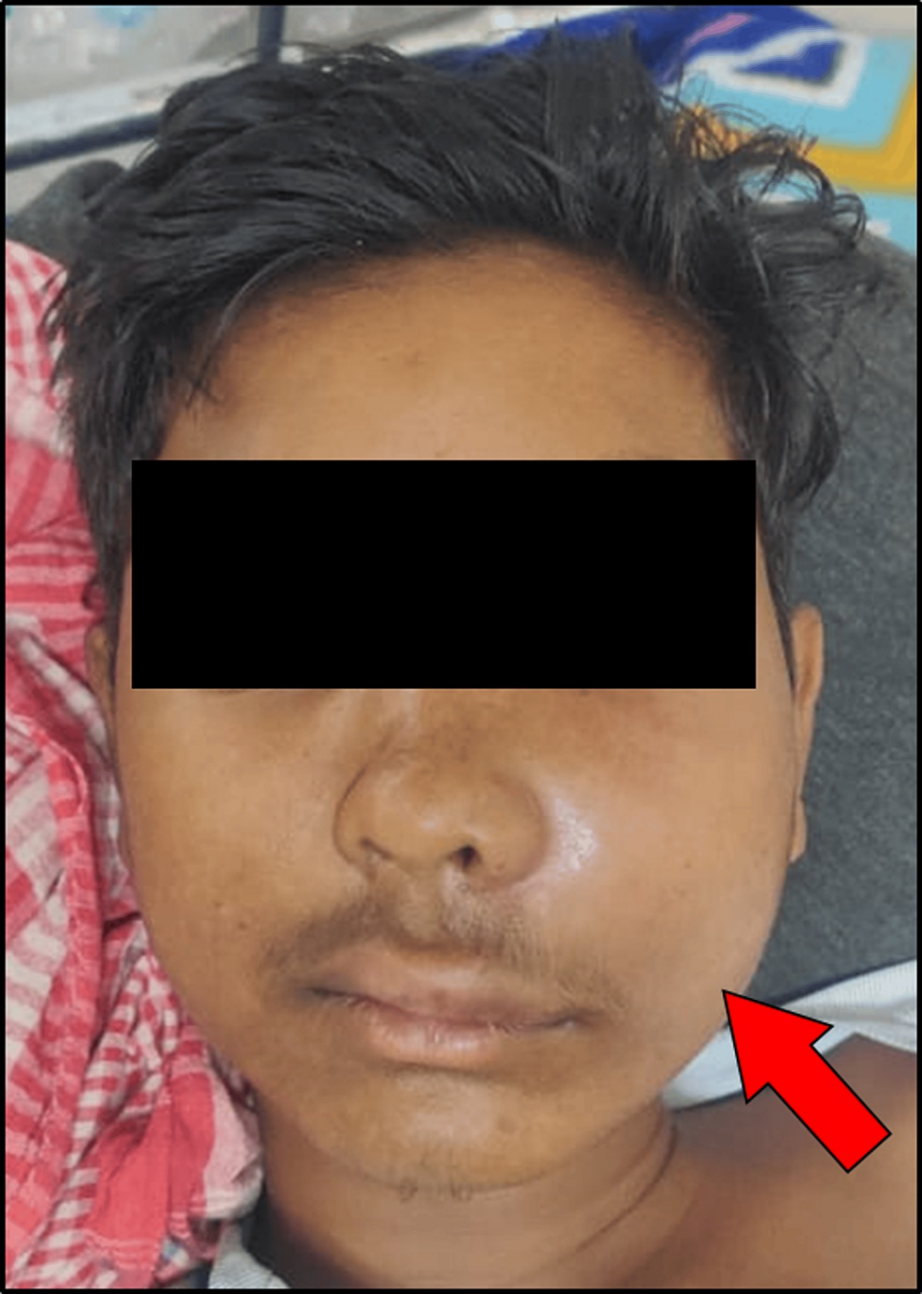
Swelling over the left malar region. The arrow depicts the presence of swelling over the left malar region, extending from the left corner of the mouth to the angle of the mandible over the left side.

A 14-year-old male was brought to the pediatrics department by his parents with the chief complaint of mild pain and diffused swelling over the left malar region (Figure 1).

Upon conducting a thorough medical history, it was noted that the patient had been diagnosed with juvenile diabetes mellitus type I, for which he was taking an insulin R injection according to a sliding scale. The patient also reported a history of trauma resulting from the mobile phone falling while being used in a supine position one month ago. Following the incident, the patient experienced persistent swelling in the left malar region, which may indicate the onset of mucormycosis. The injury from the mobile phone's fall could have potentially triggered an infection in the affected area, particularly considering the patient's weakened immune system and heightened susceptibility to opportunistic fungal infections due to diabetes. While doing routine blood investigations, the following values were observed and presented in Table 1.

**Table 1:** Observed values during routine blood investigations. These tests were conducted using a fully automated analyzer, the Vitros 5600 (Ortho Clinical Diagnostics, Raritan, New Jersey). NGSP: National Glycohemoglobin Standardization Program; ADA: American Diabetes Association.

Investigations	Observed Value	Normal Value
Random blood sugar	546 mg%	70–150 mg%
HbA1C	11.6 % A1C NGSP	Non-diabetic: <6.0% A1C NGSP
		Action suggested: >7.0% A1C NGSP
		ADA target: 6.0%–7.0% A1C NGSP

**Figure 2:**
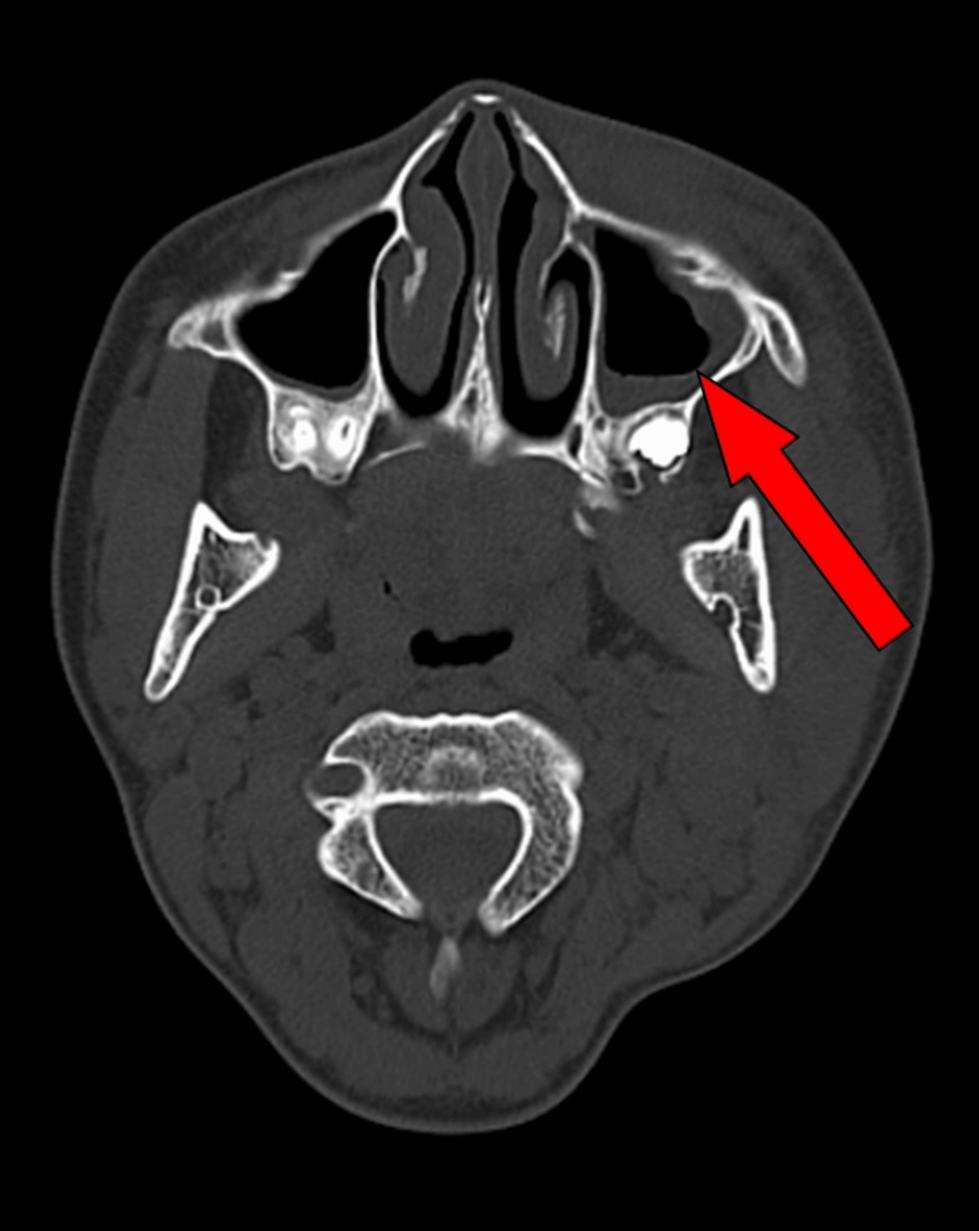
The axial section of the computed tomography scan showing the maxillary sinus. The arrow shows the thickening of the maxillary sinus walls on the left side.

The patient was maintained on an insulin infusion. The pediatrician consulted with oral and maxillofacial surgeons regarding the swelling in the left malar region. Subsequently, an ultrasound was performed on the swollen area over the left malar region, which was suggestive of an intramuscular pre-abscess (masseteric abscess). The measured dimensions of the affected muscle were 2.89 x 1.48 x 1.55 cm, with a volume of 3-5 cc. Hence, a contrast-enhanced computed tomography scan of the head was conducted, which was suggestive of maxillary sinusitis without any evidence of bony erosion (Figure 2).

**Figure 3:**
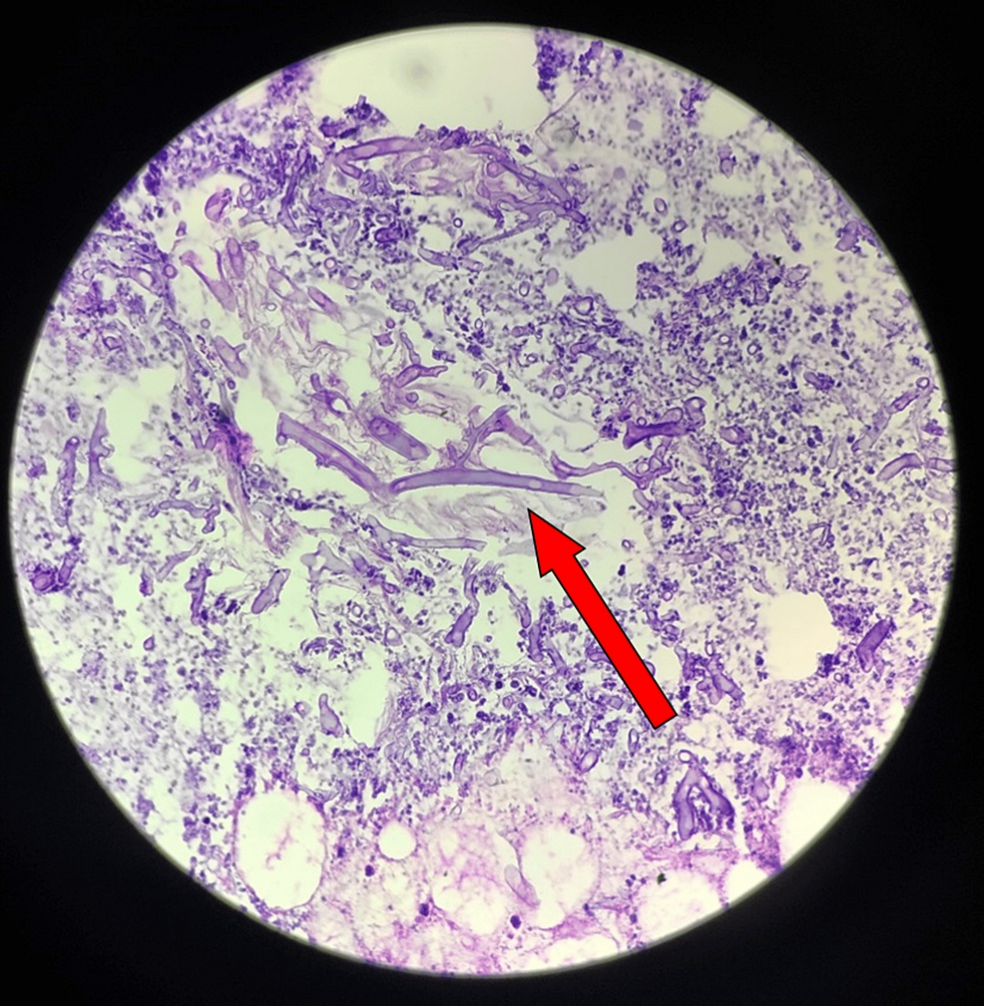
A section from the given tissue specimen shows broad, non-septate hyphae in a branching pattern. Additionally, there are features of necrosis with acute and chronic inflammatory infiltrates. Impression: the histopathological features are suggestive of mucormycosis.

We performed an aspiration procedure on the swelling using a wide-bore needle, and frank pus was aspirated. The patient was posted for an incision and drainage procedure of the abscess. Under all aseptic precautions, a left infra-orbital nerve block was administered. A stab incision was made in the left maxillary canine region intra-orally, and the area was explored using artery forceps. Approximately 18-20 mL of pus was drained and sent for microbiological examination, which was suggestive of the growth of *Klebsiella* species. The granulation tissue was sent for histopathological examination, which indicated a diagnosis of "mucormycosis" (Figure 3).

The patient is scheduled to undergo surgical debridement of the left hemi-maxilla, followed by the administration of antifungal drugs and antibiotics based on the antibiotic sensitivity report. Until the antibiotic sensitivity report is available, we cannot prescribe antifungal drugs or plan any further interventions. However, the patient's parents decided to discharge him against medical advice due to unexplained reasons. The patient did not report for follow-up despite attempts to contact his parents via phone calls.

## Discussion

Immunocompromised people are susceptible to opportunistic infections such as mucormycosis, but they can affect healthy individuals as well. According to Jones et al. (1993), patients with uncontrolled diabetes, malignancies, leukemia, glucocorticoid therapy, immunosuppressive therapy, malnutrition, and AIDS are at greater risk for mucormycosis [4]. The primary risk factors for this fungal infection in the pediatric population are hematopoietic stem cell transplantation, solid organ transplantation, and hematological malignancies (46%) [5]. Our patient, diagnosed with juvenile type 1 diabetes, suffered from mucormycosis of the maxilla. This fungus generally originates from the paranasal sinuses and disrupts the lamina papyracea, leading to orbital and maxilla involvement. The *Mucor* hypha forms a thrombus that disrupts the blood supply, causing ischemia of the site and leading to necrosis of the bone and soft tissues. According to the 2005 analysis conducted by Kulkarni et al., rhinocerebral mucormycosis predominantly affects individuals suffering from diabetes mellitus [6].

The multifactorial effects of hyperglycemia and ketoacidosis may be the cause of the association between diabetes and mucormycosis. Fungal growth is facilitated by the use and production of ketoreductase, which is aided by ketones. Other underlying conditions that were noted in the case reported by Bavikar and Mehta in 2017 included prior trauma, deferoxamine therapy, iron overload, and steroid therapy [7]. Particularly in the early stages, the initial clinical presentation may resemble bacterial cellulitis [8,9]. A fungal infection can spread to the orbital structures, which could result in proptosis, chemosis, and, in severe situations, ophthalmoplegia and even blindness. Affected sensorium, hemiparesis, orbito-facial cellulitis, headache, fever, and black nasal eschar were among the clinical findings. To evaluate this condition, the preferred diagnostic tools include CT scans, which reveal hyper-dense areas in the sinus and bone erosion. Nasal scrapings and fine-needle aspiration cytology (FNAC) are also used for the diagnosis of mucormycosis. Diabetes mellitus alters phagocytic ability with an altered polymorph nuclear leucocyte response. *Rhizopus* species thrive on a glucose-rich medium with low oxygen tension, which is common in diabetes.

Treatment includes the use of amphotericin B 50 mg every day. Some studies suggest 1 mg/kg of amphotericin B. Surgical resection is the last resort when therapeutic treatment fails to treat the patient. This includes resection of the maxilla or maxillectomy, followed by the administration of amphotericin B [10].

## Conclusions

This is a case of maxillary mucormycosis in a child with juvenile type I diabetes mellitus in the Maharashtra region. In order to effectively manage this invasive fungal disease and initiate appropriate therapy in a timely manner, a rapid and sophisticated diagnostic approach is essential.
